# Effectiveness of removals of the invasive lionfish: how many dives are needed to deplete a reef?

**DOI:** 10.7717/peerj.3043

**Published:** 2017-02-23

**Authors:** Paolo Usseglio, Jason D. Selwyn, Alan M. Downey-Wall, J. Derek Hogan

**Affiliations:** 1 Fundación In-Nova Castilla la Mancha, Spain; 2 HoBi Lab, Department of Life Sciences, Texas A&M University—Corpus Christi, Corpus Christi, TX, United States; 3 Marine Science Center, Northeastern University, Nahant, MA, United States

**Keywords:** Invasive species, Lionfish, Caribbean, Removal efficiency, *Pterois volitans*, Management prioritization

## Abstract

Introduced Indo-Pacific red lionfish (*Pterois volitans/miles*) have spread throughout the greater Caribbean and are associated with a number of negative impacts on reef ecosystems. Human interventions, in the form of culling activities, are becoming common to reduce their numbers and mitigate the negative effects associated with the invasion. However, marine managers must often decide how to best allocate limited resources. Previous work has identified the population size thresholds needed to limit the negative impacts of lionfish. Here we develop a framework that allows managers to predict the removal effort required to achieve specific targets (represented as the percent of lionfish remaining on the reef). We found an important trade-off between time spent removing and achieving an increasingly smaller lionfish density. The model used in our suggested framework requires relatively little data to parameterize, allowing its use with already existing data, permitting managers to tailor their culling strategy to maximize efficiency and rate of success.

## Introduction

Indo-Pacific red lionfish (*Pterois volitans/miles*) were introduced to South Florida in the 1980’s and have since spread throughout the greater Caribbean showing exponential population growth throughout the invaded range (Betancur-R et al., 2011), with populations in the invaded range reaching greater densities than those of their native range (Kulbicki et al., 2012). These population booms have resulted in reduced abundance of native species (Morris & Akins, 2009; Barbour et al., 2010; Côté & Maljkovic, 2010), decreases in recruitment (Albins & Hixon, 2008), and possible shifts in benthic community composition (Lesser & Slattery, 2011; Albins & Hixon, 2013; Layman, Jud & Nichols, 2014). Human intervention, in the form of culling, has become common to mitigate lionfish’s negative effects (Morris, 2012).

Total eradication of lionfish across the invaded range is unlikely due to their high abundance, wide-spread distribution and the high resilience of the species (Morris, Shertzer & Rice, 2010; Barbour et al., 2011; Arias-González et al., 2011). However, there is evidence that local-scale removal efforts can reduce lionfish abundance and have benefits for the local native reef community (Morris & Whitfield, 2009; Barbour et al., 2011; Frazer et al., 2012). In natural systems, the severity of the deleterious lionfish effects appear to be system or location-specific (Elise et al., 2015; Albins, 2015), suggesting that reef susceptibility to the negative lionfish effects may be driven by a complex number of locally determined factors, including community structure, complexity, and overall ecosystem health. Overall reef health and initial lionfish density both affect native reef resiliency and thus the necessary removal effort required to mitigate predation-induced declines of native species (Green et al., 2014). Importantly, Green et al. (2014) found that complete eradication of lionfish was not necessary for native species recovery, suggesting that culling of lionfish may be a practical solution to mitigate their impacts.

While spatially restricted culling can effectively reduce lionfish density, there is a lack of key metrics, such as the relationship between effort and percent of the population removed to assess the efficiency of this approach (Frazer et al., 2012). Marine resource managers in the Caribbean, working under budget limitations, would greatly benefit from these metrics as it would be possible to know the effort needed to achieve specific removal targets. A framework to determine the fishing effort required to achieve reductions in lionfish densities, consistent with threshold levels estimated by the model proposed by Green et al. (2014), would be a useful management tool. Here we aim to develop such a framework to aid in the effective and efficient culling of lionfish.

To estimate the effort required to reduce lionfish populations by a given percentage, as specified by the difference between the initial and target densities, we followed a three-step approach. First, we culled lionfish populations at multiple sites by conducting removal dives over consecutive days and monitored changes in catch per unit effort at each site over time. Second, we used a depletion model and the aforementioned data to estimate initial lionfish population sizes and lionfish catchability at each site. Finally, we integrated the depletion model results from all sites into a simple exponential model to determine the percentage of the initial population removed for a given amount of effort. Our proposed framework can be easily implemented using data already collected in common culling efforts and as such could be incorporated into existing removal strategies.

## Methods

### Study site

**Figure 1:**
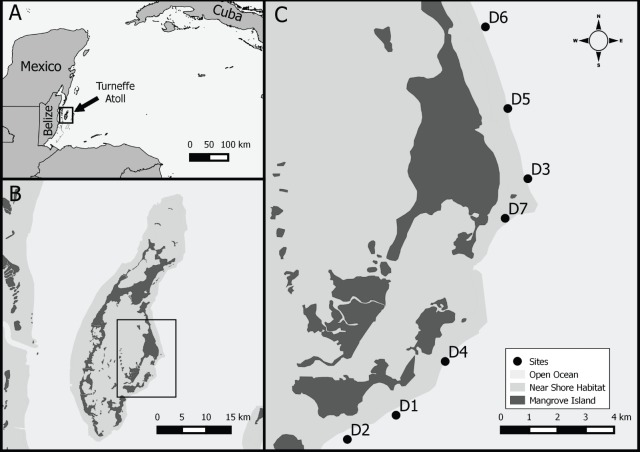
Study location. Study area, (A) Western Caribbean with location of Belize and Turneffe atoll, (B) Turneffe atoll, (C) study sites in southeast windward location on Turneffe Atoll.

We sampled seven sites on Turneffe atoll (17.3638°N, 87.8581°W), Belize, Central America, (Fig. 1). Turneffe atoll is located 9–23 km offshore of the main Belizean Barrier Reef, the largest barrier reef in the Caribbean and second largest globally (Gibson & Carter, 2003). The atoll consists of a number of mangrove islands which provide important nursery habitat for a variety of fish species, including lionfish (Gibson & Carter, 2003; Mumby, 2006; Claydon, Calosso & Traiger, 2012). The perimeter of the atoll consists of a barrier reef that transitions into a drop-off (Chittaro et al., 2006). Numerous reef patches are found within the reef lagoon. The northern portion of the windward side consists of spur and groove habitat while the southern portion is composed of drop-off and wall habitats starting at a depth of ∼18 m. A more detailed description of the area can be found in (Garcia & Holtermann, 1998).

Sampling sites were located along the outer edge of the windward forereef in the central portion of the atoll, ranging in depth from 15–30 m and area from 1,148 to 1,800 m^2^ (1,643 (±238 s.d.) m^2^, Fig. 1, Table 1). Similar to much of the Caribbean, sampling sites generally have low coral cover (4–19%), with some evidence of a coral to algal dominated community shift (McClanahan & Muthiga, 1998; Wild, Jantzen & Kremb, 2014). Between the reef crest and wall habitats, along the windward side, the reef is dominated by numerous *Orbicella* colonies.

**Table 1:** Site descriptions. Descriptions of seven experimental reef sites at Turneffe atoll, Belize, including size (m^2^), depth range (m), the mean and total number of diver-hours, the number of depletion 2 dives, the total number of lionfish caught, and whether or not we achieved our depletion criterion of two consecutive dives with zero lionfish caught (only the first of which counted towards the number of depletion dives).

Site	Area (m^2^)	Depth range (m)	Average diver hours per dive (±s.d.)	Total diver hours	Number of depletion dives	Number of lionfish caught	Depletion criterion achieved
D1	1,184	20–30	1.02 (±0.17)	5.10	5	35	Yes
D2	1,800	15–30	1.00 (±0.28)	7.03	7	43	Yes
D3	1,470	20–30	1.07 (±0.05)	4.27	4	17	No
D4	1,800	15–30	1.05 (±0.40)	5.23	5	27	Yes
D5	1,800	15–30	0.98 (±0.38)	5.85	6	32	No
D6	1,800	19–35	0.94 (±0.29)	5.67	6	32	Yes
D7	1,650	20–35	1.44 (±0.20)	8.65	6	23	No

Sampling sites were haphazardly located using natural breaks, such as sand channels, as site boundaries (Fig. 1). Additional marker buoys were placed at the corners of each site to act as guides and help minimize the accidental inclusion of offsite lionfish. Site area was measured using fiberglass tape measures.

### Depletion fishing

Divers on SCUBA removed lionfish from each site using pole-spears. For each dive, the number of divers, the time spent hunting for lionfish, the total number of lionfish, and the total length (TL, cm) and mass (g) of each lionfish were recorded. Fishing effort was calculated as diver-hours per dive, the sum of time spent by all divers hunting lionfish at each site. For example, three divers hunting lionfish during a 20-min dive would equal one diver-hour. Each site was depleted by repetitively diving the area, over successive days, until no lionfish were observed or caught on consecutive dives. As the areas of the sample sites varied substantially, all dive times and catches were standardized to diver-hours per 1,000 m^2^ and catch per 1,000 m^2^, respectively. The effect of depletion fishing on abundance was estimated by calculating catch per unit effort (CPUE) as:

(1) \begin{eqnarray*}CPUE= \frac{C}{h} ;\end{eqnarray*}

where *C* is the catch per dive per 1,000 m^2^ and h is the fishing effort expressed in diver-hours per 1,000 m^2^. The use of diver-hours per 1,000 m^2^ as the metric of effort assumes no change in catch efficiency with increased numbers of divers, for example if divers communicate to help find fish.

### Depletion model

We used a Leslie depletion model (Leslie & Davis, 1939) to estimate the initial population size per 1,000 m^2^ on all sites. A depletion model observes how the removal of fish affects the relative abundance of fish remaining in the population (Hilborn & Walters, 1992), assuming that repeated fishing over a small area will reduce local population size (Ogle, 2016a).

Initial population size is calculated as:

(2) \begin{eqnarray*}{N}_{0}=\sum _{t=1}^{T}{n}_{t};\end{eqnarray*}

where *N*_0_ is the initial population size per 1,000 m^2^, *T* is the number of removal dives and is indexed by *t*, and *n*_*t*_ is the catch per 1,000 m^2^ from the *t*-th removal.

The model assumes that catch per unit effort in the *t*-th removal event is proportional to the extant population at the time of the *t*-th removal event:

(3) \begin{eqnarray*}{CPUE}_{t}=q{N}_{t};\end{eqnarray*}

where *CPUE*_*t*_ is the catch per unit effort, *N*_*t*_ is the population size per 1,000 m^2^ for the *t*-th removal, and *q* is the catchability coefficient representing the fraction of the population that is removed by one unit of fishing effort.

The Leslie depletion model is in the form of a linear equation:

(4) \begin{eqnarray*}{CPUE}_{t}=q{N}_{0}-q{K}_{t-1};\end{eqnarray*}

where *CPUE*_*t*_ is the response variable, and *K*_*t*−1_ is the cumulative catch up to time *t* − 1, *q* is the slope and *qN*_0_ is the intercept (Leslie & Davis, 1939; Ogle, 2016a). The initial number of lionfish is thus estimated by dividing the intercept by the catchability coefficient *q*. Confidence intervals for estimates of *qN*_0_ and *q* were derived from the regression results following the methods outlined in (Ogle, 2016a).

The model assumes populations are closed to migration over the sampling period, catchability is constant through time and among individuals, enough fish are removed to substantially reduce CPUE, and the catch removes more than 2% of the population (Ogle, 2016a). We attempted to meet these assumptions by conducting depletion dives on consecutive days, with the same observers, and whenever possible, continuing depletion until zero fish were caught or seen on two consecutive dives. While some fish can exhibit fleeing behavior in response to depletion fishing activities (Giddens et al., 2014), and this has been documented for lionfish (Côté et al., 2014), we saw no such change in lionfish behavior during the course of our dives. This is likely a reflection of high capture efficiency, preventing wounded individuals from learning to avoid diver activity. Additionally, the depletion models for all sites met the standard assumptions of normality and homoscedasticity for linear regression (Figs. S1 and S2). This approach does not assume similarity in catchability among sites, or any other site characteristic, as the initial number of lionfish at each site was estimated using only measurements from that site. Despite this, there may be a relationship between the initial density of lionfish at a site and the catchability coefficient at that site due to, for example, handling time being of relatively greater importance at higher densities. To determine if the estimated initial lionfish density affected the catchability coefficient we performed a linear regression.

### Lionfish caught versus dive time

Using the estimated initial population size per 1,000 m^2^ we modeled the percentage of lionfish caught as a function of cumulative dive time per 1,000 m^2^ using an exponential asymptotic growth model following the formula:

(5) \begin{eqnarray*}C=100 \left( 1-{e}^{-\alpha qt} \right) \end{eqnarray*}

where *α* is the marginal reduction in CPUE, t is the cumulative number of diver-hours per 1,000 m^2^, *q* is the site-specific catchability coefficient, and *C* is cumulative percentage of lionfish caught at time *t*. Models were built both including and not including the catchability coefficient to test the hypothesis that catchability interacts with dive-time to affect the cumulative percentage of lionfish caught. Including catchability coefficient as a multiplier of time acts as a site-specific scalar of effort in order to standardize effort across sites with disparate degrees of removal efficacy. To account for potential variation among sites (beyond what is captured by the catchability coefficient) in coral cover or habitat complexity, for example, this function was modeled to include site as a random factor influencing *α*. The best model was chosen based on minimization of the bias corrected Akaike Information Criteria (AICc) (Akaike, 1973; Arnold, 2010), which maximizes model fit while avoiding overfitting by incorporating the log-likelihood of the model, while penalizing for the number of parameters and the small sample size. The best-fit model was used to predict the time required to remove 50% and 90% of the estimated total population of lionfish per 1,000 m^2^ based on the mean catchability coefficient.

All of the above analyses were performed with the statistical package R, version 3.0.2 (R Development Core Team, 2013), depletion models were calculated with the FSA package (Ogle, 2016b), exponential asymptotic growth models were calculated using the nlme package (Pinheiro et al., 2013), and graphing was done using the package ggplot2 (Wickham, 2009). All relevant data, analysis, and code are provided in the supplemental material (Data S1 and Supplemental Information 1).

This research was conducted under permission 000033-14 from the Belize Fisheries Department. Animal use for this project received approval by the Animal Care & Use Committee, Texas A&M University—Corpus Christi, protocol number #05-14.

## Results

### Depletion fishing

Across all seven sites, divers captured 209 lionfish, ranging in size from 12–41 cm TL (mean = 25.9 (±5.9 s.d.) cm). The total number of fish captured at each site ranged from 17 to 43 lionfish (Table 1). In four of seven sites, we were unable to achieve our depletion criterion (i.e., no lionfish observed on two consecutive dives; Table 1).

### Depletion model

**Figure 2:**
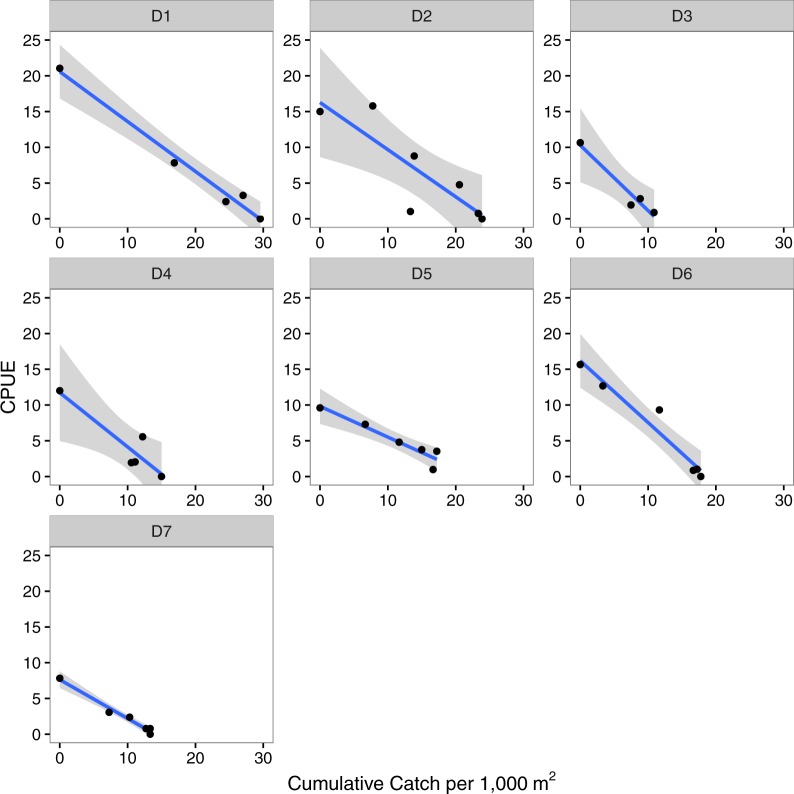
Depletion models. Leslie depletion models relating catch per unit effort (CPUE) to cumulative catch per 1,000 m^2^ that were used to estimate the initial number of lionfish present per 1,000 m^2^ (*N*_0_) and catchability coefficient (*q*) at each site (D1–D7). The line and shaded area are the regression line and 95% confidence intervals.

Leslie depletion models independently estimated the initial number of lionfish present as 11 to 29 individuals per 1,000 m^2^ per site (mean = 19.5 (±6.5 s.d.) per 1,000 m^−2^, Table 2, Fig. 2). The catchability coefficient was found to range from 0.43 to 0.91 (mean = 0.694 (±0.170 s.d.), Table 2, Fig. 2) with higher values indicating lionfish were easier to capture at a particular site. The models’ *r*^2^ values ranged from 0.68–0.98 and were all significant, suggesting a good fit of the data to the model (Table 2). There was no effect of the initial estimated density of lionfish on the catchability coefficient (*r*^2^ = 0.13, *p* = 0.45; Fig. S3A).

**Table 2:** Depletion model results. Summary of results of the depletion diving and estimating initial lionfish abundance based on the Leslie depletion model (Eq. 4; Fig. 2). The initial number of lionfish (*N*_0_), catchability coefficient (*q*), coefficient of determination (*r*^2^), and *p*-value (*p*) are shown for each site (D1–D7). Numbers in parentheses represent 95% confidence intervals of estimates.

Site	*N*_0_	*q*	*r*^2^	*p*
D1	29 (26–33)	0.70 (0.53–0.87)	0.98	0.0009
D2	25 (16–34)	0.66 (0.20–1.11)	0.68	0.0137
D3	11 (7–16)	0.91 (0.26–1.56)	0.92	0.0262
D4	15 (9–22)	0.76 (0.15–1.37)	0.78	0.0291
D5	23 (17–29)	0.43 (0.24–0.62)	0.88	0.0035
D6	19 (15–22)	0.86 (0.58–1.15)	0.93	0.0011
D7	14 (13–15)	0.54 (0.43–0.65)	0.97	0.0001

### Lionfish caught versus dive time

**Figure 3:**
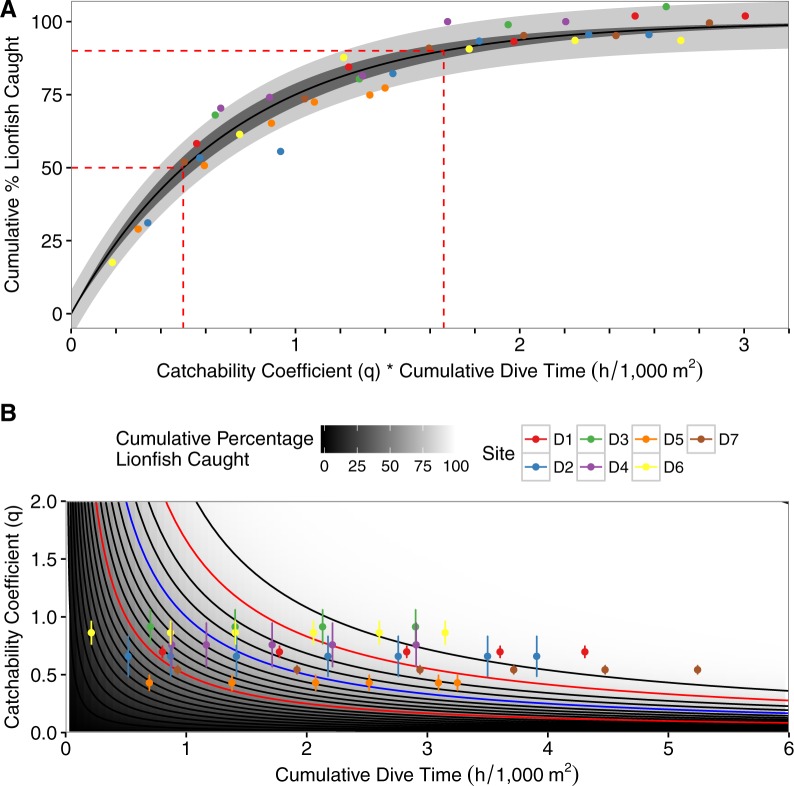
Asymptotic exponential growth model. (A) Plot of asymptotic exponential growth model with percent removal of lionfish per 1,000 m^2^ vs. the product of dive-time per 1,000 m^2^ and the catchability coefficient. Dashed red lines represent time needed to remove 50% and 90% of the population, the darkly shaded area represents the 95% confidence interval, the lighter shaded area represents the 95% prediction interval, and colored points represent different depletion sites (D1–D7). (B) Contour plot of the results of the best-fit model of percent removal of lionfish per 1,000 m^2^ based on dive-time per 1,000 m^2^ and catchability coefficient (Eq. 4). Points represent observed values with standard deviations, and colored points represent different depletion sites (D1–D7). Contours represent the cumulative percent of lionfish caught and increment by 5% with darker colors being closer to 0% and lighter colors closer to 100%. Red contours are the 50% and 90% contour lines and the blue contour is at 75% removal, as used in the theoretical example.

The best-fit model incorporated among-site variability as a random factor affecting *α* and the interaction between the catchability coefficient and dive-time (Eq. 6; Table 3; Fig. 3). The model predicted, with a 95% CI, that one hour of fishing effort per 1,000 m^2^, scaled using the mean catchability coefficient (0.69), results in a 58%–66% reduction in the lionfish population per 1,000 m^2^. Four diver-hours per 1,000 m^2^, scaled by the mean catchability coefficient, achieves 97%–99% reductions of the initial population per 1,000 m^2^ (Fig. 3A). The percent reduction of lionfish for a specified amount of effort is inversely related to the catchability coefficient (Fig. 3B). As such when one hour of fishing effort per 1,000 m^2^ is scaled based on the minimum observed catchability coefficient (0.43) there is a 41%–49% reduction in lionfish; when scaled with the maximum catchability coefficient (0.91) there is a 68%–76% reduction in lionfish (Fig. 3B). Similarly, a 50% decrease of the initial population size was achieved after between 1.16–1.17 (minimum observed catchability) to 0.54–0.55 (maximum observed catchability) diver-hours per 1,000 m^2^depending on the catchability coefficient (0.71–0.72 diver-hours per 1,000 m^2^ based on the mean catchability coefficient). A 90% reduction required between 3.83–3.88 (minimum observed catchability) to 1.80–1.83 (maximum observed catchability) diver-hours per 1,000 m^2^ depending on the catchability coefficient (2.37–2.41 diver-hours per 1,000 m^2^ based on the mean catchability coefficient; Fig. 3B).

**Table 3:** Lionfish catch as a function of cumulative dive time. Comparison of the non-linear mixed effects models of cumulative lionfish catch as a function of cumulative dive time. Scaling indicates whether the cumulative dive time was scaled by the catchability coefficient (indicated as q-scaled). Random indicates which models included site as a random factor influencing the multiplication coefficient *α*. Parameter estimates (±SE) are shown based on each model. *α* is the coefficient in the exponential models multiplied by cumulative dive time, or catchability coefficient (*q*) and cumulative dive time. *K* is the number of parameters estimated in the model. logLik is the log likelihood of the model and ΔAICc is the difference in AICc from the best model

Model	Scaling	Random	*α*	Intercept	*K*	logLik	AICc	Δ AIC	AICc Weight
1	q-scaled	Site	1.39 ± 0.08		3	−116.56	239.81	0.00	0.99
2	q-scaled		1.35 ± 0.05		2	−122.13	248.59	8.78	0.01
3		Site	0.99 ± 0.12		3	−122.51	251.70	11.89	0.00
4			0.92 ± 0.06		2	−147.47	299.27	59.46	0.00
Null Model				78.09 ± 3.51	2	−173.08	350.50	110.69	0.00

(6) \begin{eqnarray*}C=100 \left( 1-{e}^{-1.39qt} \right) .\end{eqnarray*}

## Discussion

Here we created a simple, yet powerful, framework for predicting the removal effort required to achieve specific lionfish removal targets (represented as the percent of lionfish remaining on the reef). We demonstrated an important trade-off between time spent removing lionfish and the density reduction. We suggest that—when coupled with a model explicitly used to determine critical lionfish densities required for native reef recovery (see the model by Green et al., 2014)—our model allows managers to create strategies that not only consider local ecosystem demographics and lionfish densities, but also the resources available (i.e., the number of available divers). This would enable managers to tailor their culling strategy to maximize efficiency and rate of success.

Generally, our model estimated a 50% reduction of lionfish in less than one diver-hour per 1,000 m^2^, and up to 90% reduction in fewer than 2.5 diver-hours per 1,000 m^2^. Given the importance of among-site variation in our model, these values should be applied cautiously, but may be applicable to similar reef systems in the western Caribbean, or used as reference values when other data is unavailable. Importantly, the results demonstrate a well-fit model that requires relatively little data to parameterize. Thus, the model provides a valuable framework for creating local or system-specific estimates for removal effort per unit area.

Variability among sites was partially accounted for within the model by including a site-specific catchability coefficient. This value reflects unquantified variables that cause catch rate to vary among sites, such as subtle changes in reef structure, rugosity, lionfish behavior, shifts in lionfish size structure, and changes in diver hunting efficiency. Furthermore, habitat structure and an adaptive behavioral response to culling (i.e., lionfish wariness) have been shown to negatively affect hunting efficiency (Green et al., 2014; Côté et al., 2014).

The initial lionfish density could affect removal time due to relatively more time spent on handling than on search at high density. Interestingly, we found no effect of initial lionfish density on the catchability coefficient. This result should be interpreted with caution as our analysis was performed over a relatively small range of densities compared to what has been observed by others (110–290 ha^−1^ this study; ∼20 ha^−1^, (Whitfield et al., 2007); 390 ha^−1^, (Green & Côté, 2009); ∼1,450 ha^−1^, (Dahl & Patterson, 2014).

The efficacy of any removal strategy is expected to be driven partially by the rate of immigration and recruitment into the managed area after a removal. In species with complex, life-stage dependent habitat utilization patterns, the size and abundance of immigrating individuals may be habitat-specific. In lionfish, several studies have found evidence of an ontogenetic shift, with new recruits and juveniles utilizing protected, shallow water habitat prior to migrating out to deeper forereefs (Barbour et al., 2010; Claydon, Calosso & Traiger, 2012; A Downey-Wall & JD Hogan, 2015, unpublished data). Consequently, management strategies that focus on removing lionfish from forereefs (i.e., this study) will likely experience the highest immigration pressure from juvenile and adult life-stage fish. Lionfish at these life stages have demonstrated an ability to move thousands of meters, but tend to exhibit comparatively small home range tendencies (Bacheler et al., 2015; Tamburello & Côté, 2015). Furthermore, lionfish immigration on patch reefs has been shown to be density independent (Benkwitt, 2013), suggesting immigration pressure will remain similar regardless of the removal area density. Overall, creating estimates for immigration rate will be essential for long-term management success, as it can drive both the frequency and intensity of removals. We suggest resolving recolonization rate, particularly at different removal areas, habitat types, and depths, should be an important priority for future research.

The framework presented here for predicting lionfish removal effort provides a starting point for management. However, there are a number of factors that can affect long-term sustainability of culling programs, the effect of which are not yet understood. For example, the size of the area to be managed, the number and experience of team members and the speed at which recolonization happens post-culling can affect costs and efficiency of removals and therefore impact sustained management. This study only investigated relatively small sites (∼1,600 m^2^) using a consistent and minimal team of experienced divers (2–3) and did not repetitively cull a single site to maintain lionfish densities as one might as part of a management strategy. It is unknown how the effort required to remove lionfish to achieve or maintain target densities will vary in response to changes in these factors. Some factors may cause non-linear changes in efficiency or costs; for example, increasing the number of divers may lead to greater improvement than expected due to diver communication, but will also cost more than increasing dive times or number of dives per personnel member.

Despite these remaining challenges, it is clear that a simple framework for lionfish culling programs can aid in management decision-making. In order to maximize the outcome of lionfish fishing activities, we suggest a framework with a series of steps that managers could follow. The first step is to survey the target reefs to assess densities of both lionfish and native species. Following Green et al. (2014), managers can use survey data as inputs into their model in order to calculate reef specific lionfish density thresholds. To determine the appropriate amount of effort required to meet those threshold densities, lionfish depletion dives can then be performed at all or some of the sensitive sites following the methods presented in this paper. Subsequent or additional removal efforts can then be planned using the relationship between diver effort and percent removal of lionfish.

As a hypothetical example, upon surveying fish populations at a site and using the model in Green et al. (2014) we found a target density of 7 lionfish/1,000 m^2^ with an initial density of 29 lionfish/1,000 m^2^ (a reduction of 75%). We would next use the model derived from the depletion dives shown here (Eq. 6) to calculate that the product of the cumulative dive time and catchability required to achieve this goal is ∼1 scaled diver-hour. Then, we divide this estimate by various catchability values to see a range of estimates based on different scenarios (e.g., inexperienced divers, particularly high complexity reef; Fig. 3B blue contour). Using the range of catchability estimates from our depletion dives (0.43–0.91, Table 2) we would then estimate that it would take between 1 and 2.3 diver hours to achieve the target density at the hypothetical site. Having this information, we can determine the optimal number of divers needed and identify specific divers with known catch rates to determine our personnel composition. This allows us to estimate the amount of fuel and boat time required to accomplish the task. These estimates are vital to agencies with limited resources as they allow for more accurate budgeting of time and money.

Controlling invasive lionfish populations has become a major objective of many agencies involved in the management of coral reefs in the tropical and sub-tropical Atlantic. Given that resources such as money, time and personnel are limited, managers need tools to effectively plan and allocate resources to achieve lionfish control objectives. Current best practices suggest targeting removal sites of particular interest: ecologically sensitive sites (such as marine protected areas) or tourism areas (Morris, 2012). Generally, it is advisable for these areas to be small to allow for tractable removal efforts with limited resources. The methods applied here are broadly applicable as a straightforward way for managers to estimate the effort needed to achieve target levels of lionfish in an area of interest. As this framework incorporates only data commonly collected during removal efforts (catch per dive and dive time), it could be implemented as part of already existing management strategies, including using previously culled sites to calculate the continued effort needed into the future. By following these steps, managers can balance their field needs with their assets in order to choose the best course of action based on the specific circumstances they face.

##  Supplemental Information

10.7717/peerj.3043/supp-1 Figure S1 Site descriptive QQ-plots with Shapiro-Wilk normality test *p*-values for each site. The blue line is the median, the green dashed line is 95% confidence interval and the red dashed line is the 99% confidence interval.

10.7717/peerj.3043/supp-2 Figure S2 Residual vs. fitted plot Residual versus fitted plot with Breusch-Pagan Test of homoscedascticity for each site.

10.7717/peerj.3043/supp-3 Figure S3 Catchability coefficient vs. estimated population size Plot of the (A) linear regression of the initial number of lionfish per 1,000 m^2^ and the catchability coefficient estimated using individual site Leslie depletion models. Each point represents a site, the blue line is the regression line and the grey ribbon is the 95% confidence interval. In the QQ-plot (B) the blue line is the median, the green dashed line is 95% confidence interval and the red dashed line is the 99% confidence interval. These data meet the assumption of normality (Shapiro-Wilk normality test *W* = 0.973, *p* = 0.921). The residual versus fitted plot (C) shows no pattern of error and the data meet the assumption of homoscedasticity (Breusch-Pagan Test BP = 0.204, *p* = 0.651).

10.7717/peerj.3043/supp-4 Figure S4 Diagnostic plots of the best fit model Diagnostic plots of the best fit model (Fig. 3, (6)). In the QQ-plot (A) the blue line is the median, the green dashed line is 95% confidence interval and the red dashed line is the 99% confidence interval. These data meet the assumption of normality (Shapiro-Wilk normality test *W* = 0.973, *p* = 0.455). The residual versus fitted plot (B) shows no pattern of error and the data meet the assumption of homoscedasticity (spline model edf = 2.61, *p* = 0.413).

10.7717/peerj.3043/supp-5 Data S1 Dataset

10.7717/peerj.3043/supp-6 Supplemental Information 1 R script for analysis R script used to predict depletion times based on initial population size.

## Additional Information and Declarations

### Field Study Permissions

The following information was supplied relating to field study approvals (i.e., approving body and any reference numbers): This research was conducted under permission 000033-14 from the Belize Fisheries Department.

### Data Availability

The following information was supplied regarding data availability: The raw data has been supplied as a Supplemental Dataset.
