# Supplementary figures and images for: Effectiveness of removals of the invasive lionfish: how many dives are needed to deplete a reef?
Source: PeerJ. 2017 Feb 23;5:e3043. doi: 10.7717/peerj.3043 (PMC5326545; doi:10.7717/peerj.3043)

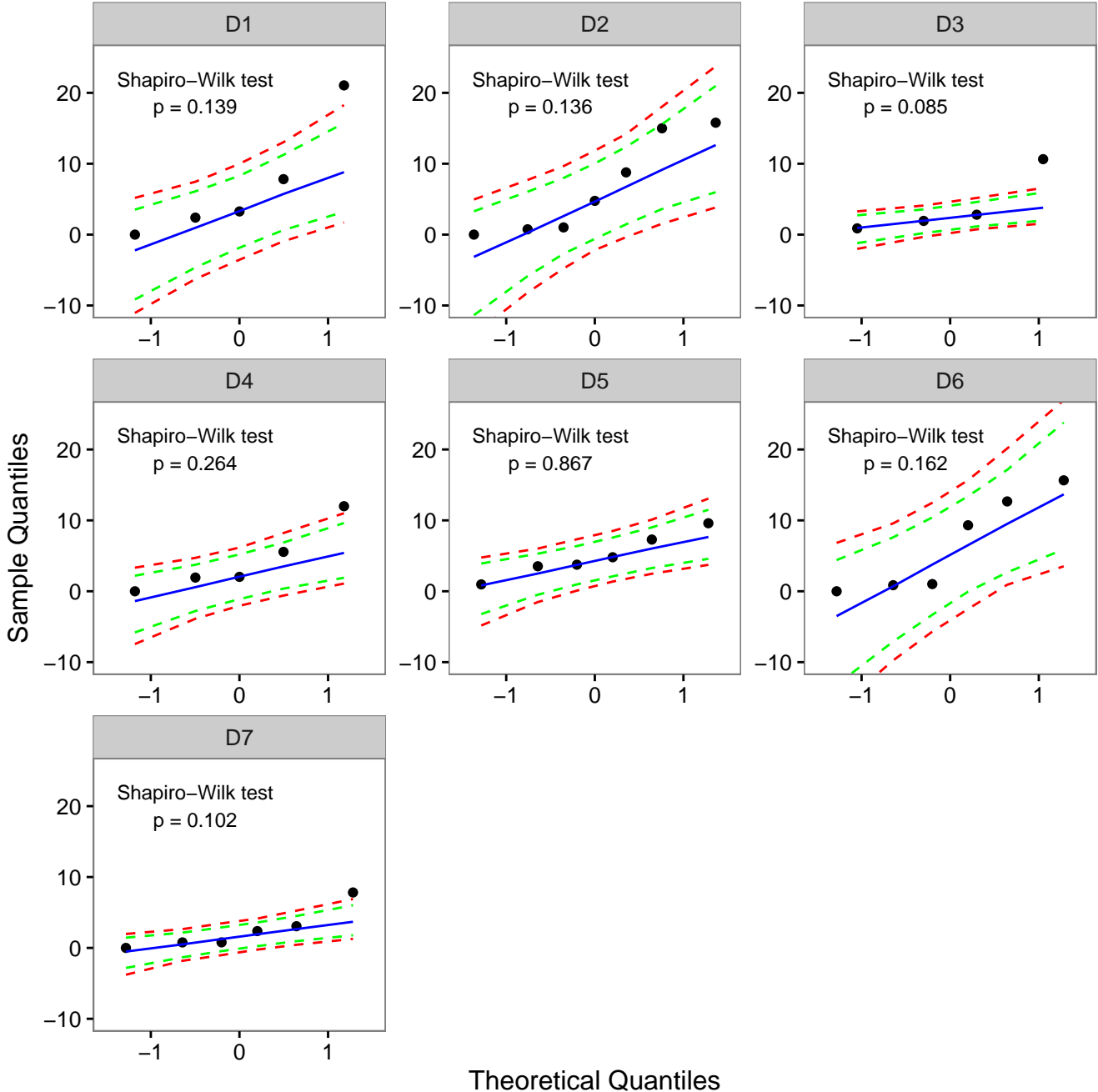

Supplement: Figure S1 — QQ-plots with Shapiro-Wilk normality test p-values for each site. The blue line is the median, the green dashed line is 95% confidence interval and the red dashed line is the 99% confidence interval. [file peerj-05-3043-s001.pdf]

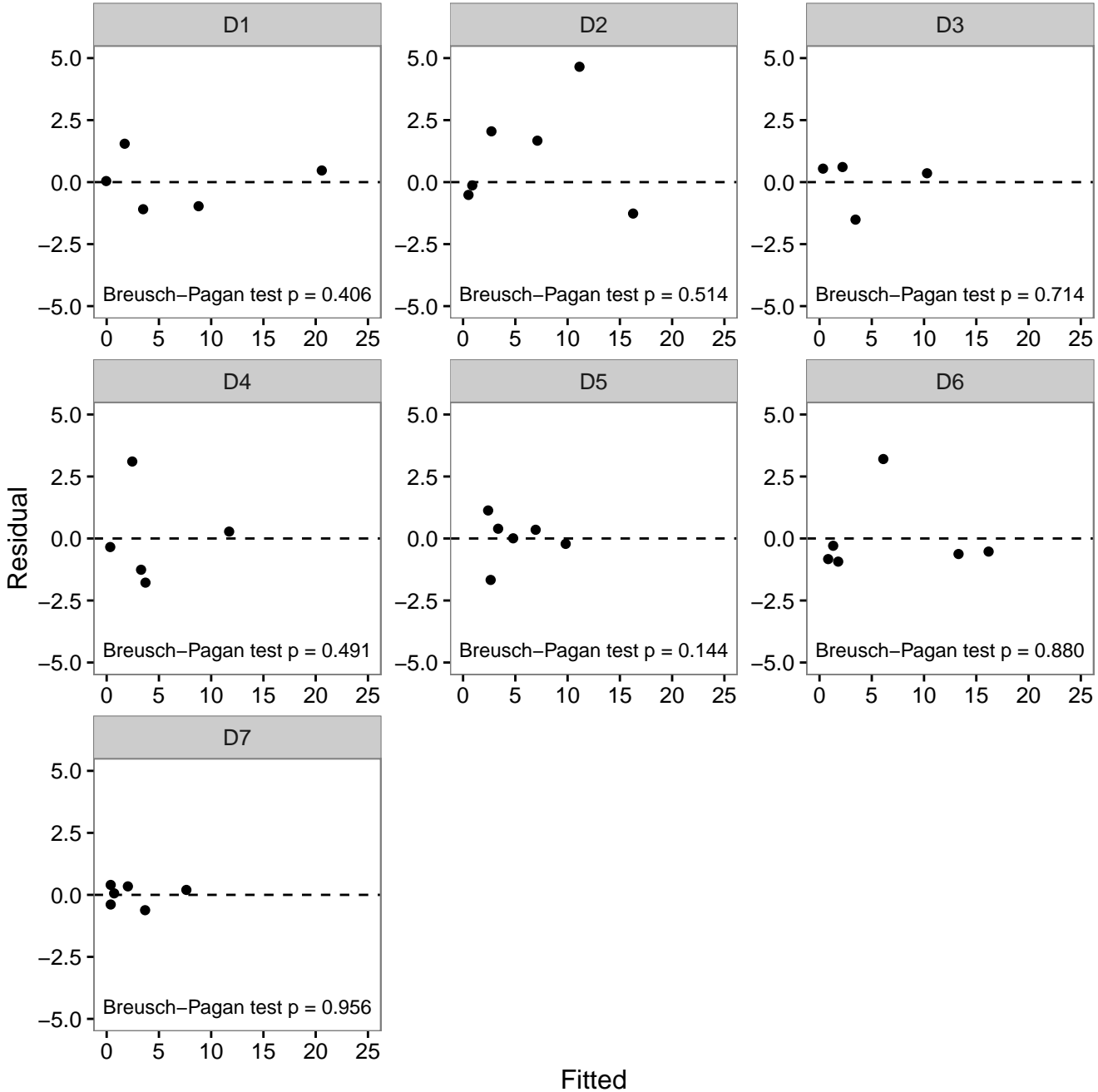

Supplement: Figure S2 — Residual versus fitted plot with Breusch-Pagan Test of homoscedascticity for each site. [file peerj-05-3043-s002.pdf]

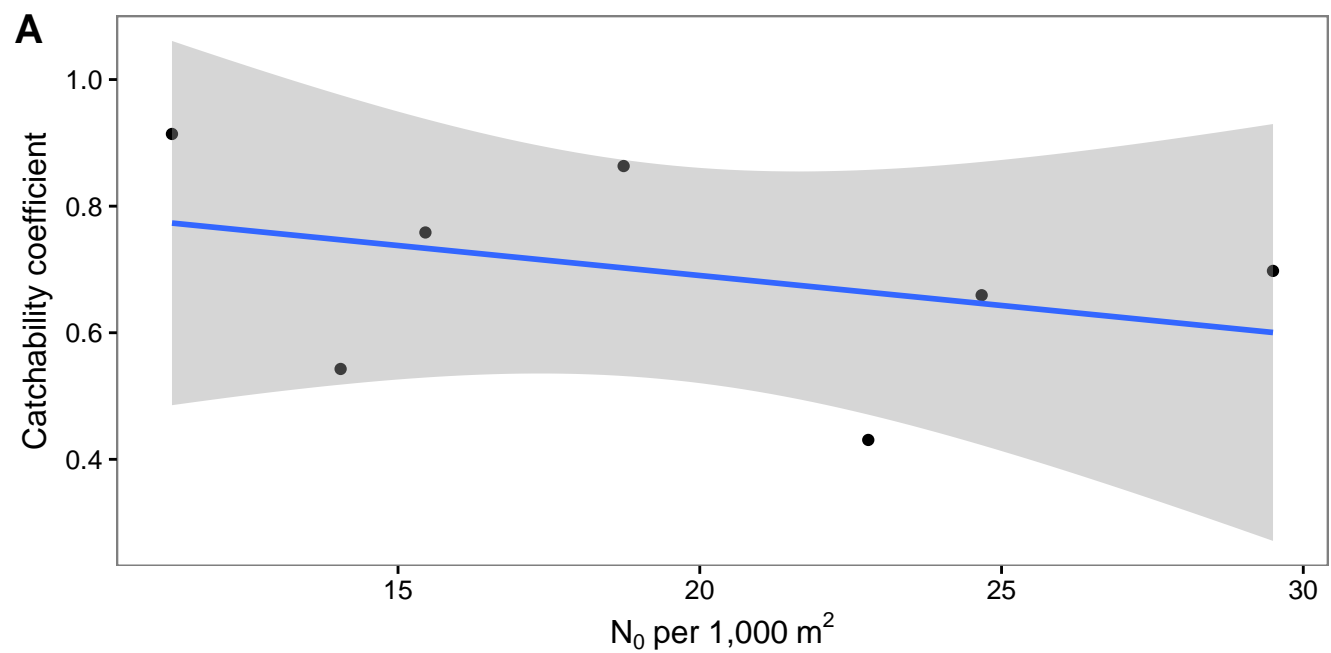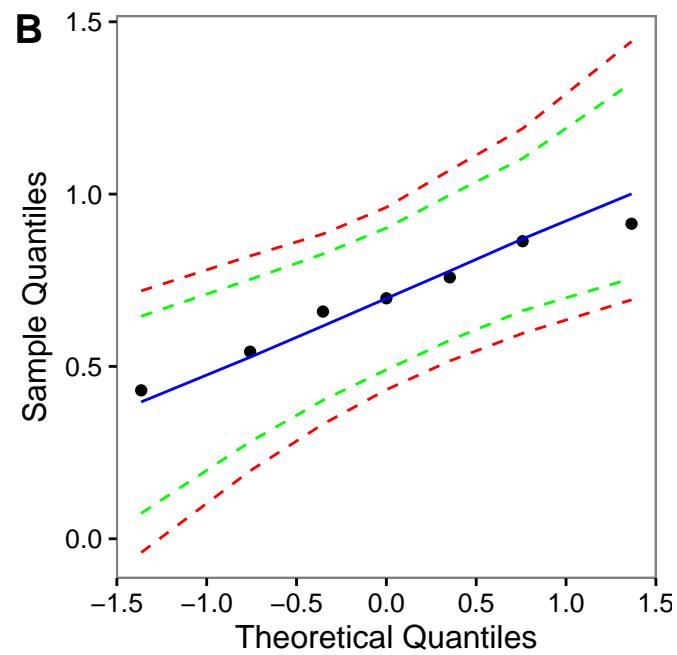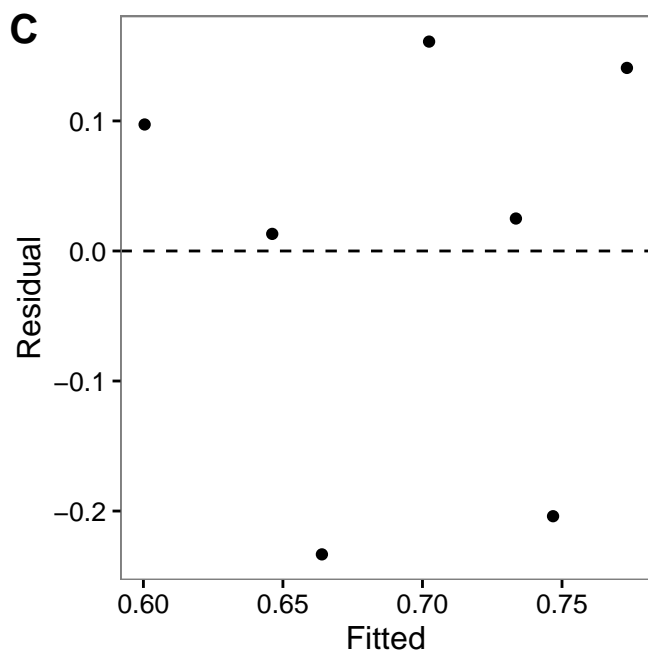

Supplement: Figure S3 — Plot of the (A) linear regression of the initial number of lionfish per 1,000 m2 and the catchability coefficient estimated using individual site Leslie depletion models. Each point represents a site, the blue line is the regression line and the grey ribbon is the 95% confidence interval. In the QQ-plot (B) the blue line is the median, the green dashed line is 95% confidence interval and the red dashed line is the 99% confidence interval. These data meet the assumption of normality (Shapiro-Wilk normality test W = 0.973, p = 0.921). The residual versus fitted plot (C) shows no pattern of error and the data meet the assumption of homoscedasticity (Breusch-Pagan Test BP = 0.204, p = 0.651). [file peerj-05-3043-s003.pdf]

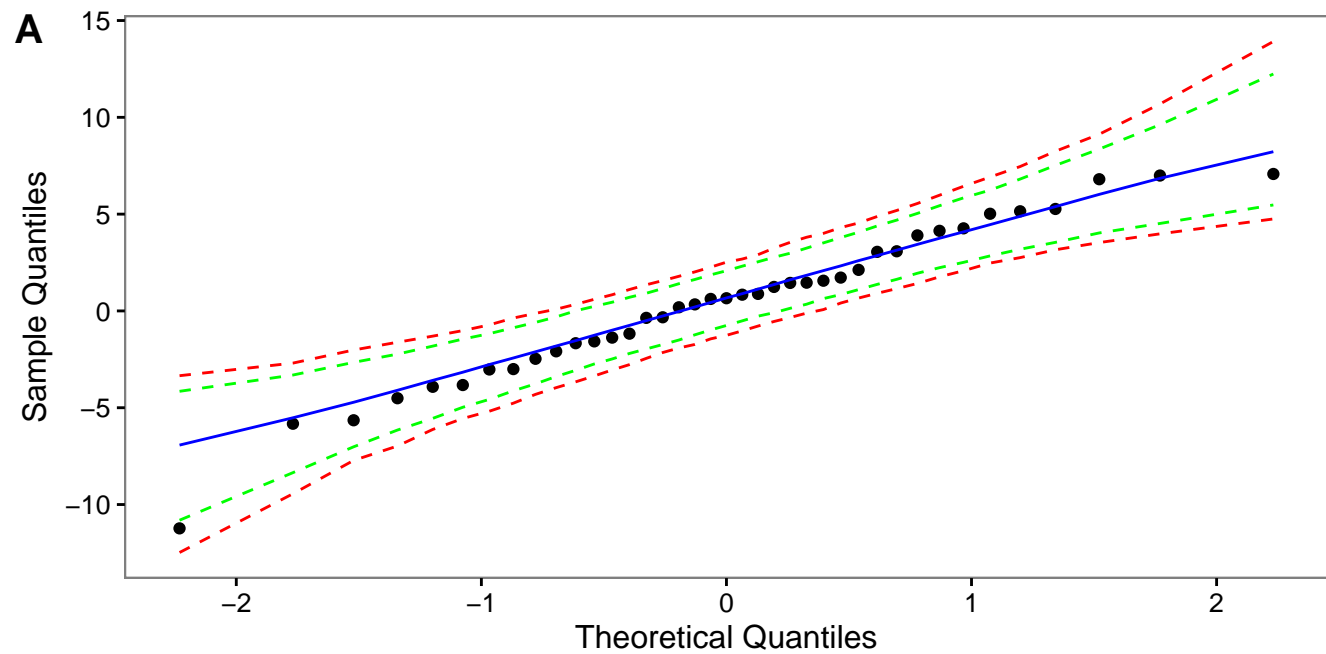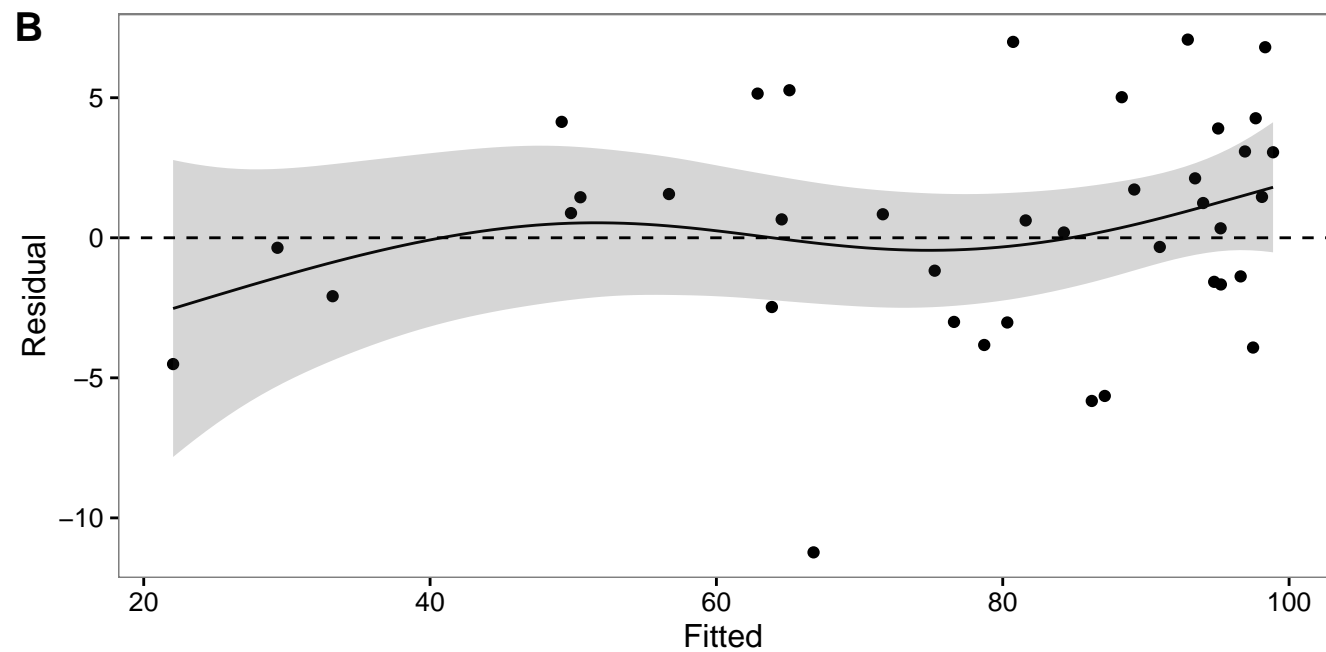

Supplement: Figure S4 — Diagnostic plots of the best fit model (Fig. 3, (6)). In the QQ-plot (A) the blue line is the median, the green dashed line is 95% confidence interval and the red dashed line is the 99% confidence interval. These data meet the assumption of normality (Shapiro-Wilk normality test W = 0.973, p = 0.455). The residual versus fitted plot (B) shows no pattern of error and the data meet the assumption of homoscedasticity (spline model edf = 2.61, p = 0.413). [file peerj-05-3043-s004.pdf]
